# Cyanotoxin Screening in BACA Culture Collection: Identification of New Cylindrospermopsin Producing Cyanobacteria

**DOI:** 10.3390/toxins13040258

**Published:** 2021-04-03

**Authors:** Rita Cordeiro, Joana Azevedo, Rúben Luz, Vitor Vasconcelos, Vítor Gonçalves, Amélia Fonseca

**Affiliations:** 1 CIBIO, Centro de Investigação em Biodiversidade e Recursos Genéticos, InBIO Laboratório Associado, Pólo dos Açores, Universidade dos Açores, 9500-321 Ponta Delgada, Portugal; ruben.fs.luz@uac.pt (R.L.); vitor.mc.goncalves@uac.pt (V.G.); maria.ao.fonseca@uac.pt (A.F.); 2 Faculdade de Ciências e Tecnologia, Universidade dos Açores, 9500-321 Ponta Delgada, Portugal; 3 Interdisciplinary Centre of Marine and Environmental Research—CIIMAR/CIMAR, University of Porto, Terminal de Cruzeiros do Porto de Leixões, Av. General Norton de Matos s/n, 4450-208 Matosinhos, Portugal; joana.azevedo@ciimar.up.pt (J.A.); vmvascon@fc.up.pt (V.V.); 4 Department of Biology, Faculty of Sciences, University of Porto, 4069-007 Porto, Portugal

**Keywords:** microcystin, saxitoxin, cylindrospermopsin, ESI-LC-MS/MS, 16S rRNA phylogeny, Azores, 16s rRNA phylogenetic analysis revealed evidence for new toxic cyanobacteria taxa, in two identified CYN producing nostocalean strains BACA0025 and BACA0031.

## Abstract

Microcystins (MCs), Saxitoxins (STXs), and Cylindrospermopsins (CYNs) are some of the more well-known cyanotoxins. Taking into consideration the impacts of cyanotoxins, many studies have focused on the identification of unknown cyanotoxin(s)-producing strains. This study aimed to screen strains from the Azorean Bank of Algae and Cyanobacteria (BACA) for MCs, STX, and CYN production. A total of 157 strains were searched for *mcy*, *sxt,* and *cyr* producing genes by PCR, toxin identification by ESI-LC-MS/MS, and cyanotoxin-producing strains morphological identification and confirmation by 16S rRNA phylogenetic analysis. Cyanotoxin-producing genes were amplified in 13 strains and four were confirmed as toxin producers by ESI-LC-MS/MS. As expected *Aphanizomenon gracile* BACA0041 was confirmed as an STX producer, with amplification of genes *sxt*A, *sxt*G, *sxt*H, and *sxt*I, and *Microcystis aeruginosa* BACA0148 as an MC-LR producer, with amplification of genes *mcy*C, *mcy*D, *mcy*E, and *mcy*G. Two nostocalean strains, BACA0025 and BACA0031, were positive for both *cyr*B and *cyr*C genes and ESI-LC-MS/MS confirmed CYN production. Although these strains morphologically resemble *Sphaerospermopsis*, the 16S rRNA phylogenetic analysis reveals that they probably belong to a new genus.

## 1. Introduction

Due to their impacts on ecosystem degradation, public health risk, and associated economical losses, cyanotoxins are one of the most studied primary and secondary metabolites from cyanobacteria [1,2,3,4]. Microcystins (MCs), Saxitoxins (STXs), and Cylindrospermopsins (CYNs) are some of the more well-known cyanotoxins, for which a high number of studies were done covering different aspects such as their taxonomic distribution, genetic organization, biosynthesis pathways, and mechanisms of action [5,6,7,8,9,10].

Microcystins are hepatotoxins produced by non-ribosomal pathways, and the most common and more well-studied cyanotoxins, with over 240 variants (e.g., MC-LR, MC-LY, MC-RR). Mostly produced by *Microcystis aeruginosa* [11], MCs are, however, currently known to be produced by over 40 species [12]. The gene cluster responsible for the biosynthesis of MCs (*mcy*) contains 10 encoding genes organized in two operons (*mcy*A-C and *mcy*D-J) in which each gene has a specific function for the toxin synthesis, regulation, and release [13,14]. This hydrophilic peptide has an Adda group in its structure, responsible for its toxicity [15,16]. With over 240 analogs, other groups as ADMAdda have been identified and characterized as responsible for MCs more toxic variants [17].

The alkaloid STX is one of the most potent natural neurotoxin [18] identified in both cyanobacteria and dinoflagellates [19]. In cyanobacteria, STXs have been identified in several species as *Aphanizomenon gracile*, *Cylindrospermum stagnale* [20], *Dolichospermum circinale* [21], and *Raphidiopsis raciborskii* [22]. The gene cluster *sxt* encodes 26 genes responsible for the biosynthesis of STXs [19] and its gene organization varies between species, which may indicate gene loss or transfer between species [6,23,24]. This scenario has been observed in the STX gene cluster where the *sxt*A gene, recognized as the encoding gene for the polyketide synthase-like structure involved in the first step of STXs production [6], was found in both STXs-producing and non-producing *A*. *gracile* strains, but all non-producing strains lost at least one of the other genes of the cluster (e.g. *sxt*G, *sxt*H, *sxt*I, or *sxt*X) [25].

Cylindrospermopsins are cytotoxic alkaloids initially identified in *Raphidiopsis raciborskii* [26] but also reported to be produced by other species as *Aphanizomenon gracile* [27] or *Chrysosporum ovalisporum* [28]. As MCs, CYNs biosynthesis is done by a non-ribosomal pathway, where the gene cluster *cyr* (15 encoding genes) has been identified as responsible for CYNs synthesis, regulation, and exportation in *R. raciborskii* [29].

Cyanobacteria have high morphological and ecological variability [30], and due to this, its taxonomical classification is relatively intricate and is in constant change [31]. New cyanobacteria families, genera, and species, as Oculatellaceae and Trichocoleaceae [32], *Aliinostoc* [33], *Halotia* [34], and *Lusitaniella* [35], and *Hyella patelloides* [35] and *Compactonostoc shennongjiaensis* [36] have been reported in recent years. These taxonomic redefinitions are challenging for the identification and distribution of toxins in many of the new genera [37], thus increasing the lack of knowledge on toxic strains. Although cyanobacteria are widely distributed through many environments [7,38], most cyanobacteria and cyanotoxins studies are focused on planktonic species from inland freshwater systems [7,20], with less information in extreme environments (e.g., hot springs, caves, hypersaline lakes, polar deserts), especially regarding their toxicity [7,39].

Taking into consideration cyanotoxins impacts, either in environmental or public health, many scientific studies have focused on the identification of unknown cyanotoxin(s)-producing species, usually by a combination of methods as immunoassays (e.g., ELISA—enzyme-linked immunosorbent assay), by targeting biosynthesis encoding genes (PCR—polymerase chain reaction) and/or by analytical methods, as liquid chromatography [40,41]. The liquid chromatography-mass spectrometry (LC-MS) is a highly sensitive and selective method for cyanotoxin detection and identification [40,41]. It can be used for cyanotoxins confirmation even in very low concentrations, as well as analog identification, even without available standards [40].

The main aim of this study was to screen for cyanotoxin-producing cyanobacteria in strains isolated from several environments (freshwater, terrestrial and thermal) from the Azores islands using a polyphasic approach, based on the detection of MCs, STX, and CYN encoding genes, the identification of their production by LC-MS, and the phylogenetic distribution of toxic strains through 16S rRNA molecular identification.

## 2. Results

### 2.1. Detection of Cyanotoxin(s)-Producing Cyanobacteria

From the 157 screened cyanobacteria strains, 13 were identified as cyanotoxin(s) potential producers by PCR amplification of the STX, CYN, and/or MC encoding genes, and four were confirmed as toxin producers by ESI-LC-MS/MS (Figures S1–S6; Table 1; Table S2).

STX producing genes were detected in six strains, however, the only strain with amplification of all the searched STX-encoding genes, *Aphanizomenon gracile* BACA0041, was the only one confirmed as saxitoxin producer by ESI-LC-MS/MS (Table 1).

CYN producing genes were detected in five strains, although only BACA0025 and BACA0031 had amplification of both searched *cyr*B and *cyr*C genes. These two strains were also the only ones with CYN detection by ESI-LC-MS/MS. The remaining three strains with positive results for the detection of CYN encoding genes only amplified the *cyr*B gene (Table 1).

MC-encoding genes were only detected in *Microcystis aeruginosa* BACA0148 (*mcy*C, *mcy*D, *mcy*E, and *mcy*G) and *Nostoc* sp. BACA0091 (*mcy*E). However, in the ESI-LC-MS/MS analysis, MC-LR ions were identified only in *M. aeruginosa* BACA0148.

### 2.2. Phylogenetic Characterization

Strains BACA0025 and BACA0031 had similarities with sequences deposited in the GenBank NCBI between 96 and 97%, which shows the distinctiveness of these strains, while strains BACA0041 and BACA0148 had similarities greater than 98% with *Aphanizomenon gracile* and *Microcystis aeruginosa* strains, respectively (Table S3). In the 16S rRNA phylogenetic tree strains BACA0041 and BACA0148 are positioned in clades with strains close to their initial identification, while strains BACA0025 and BACA0031 had high phylogenetic distances from the closest morphological identified strains.

**Figure 1:**
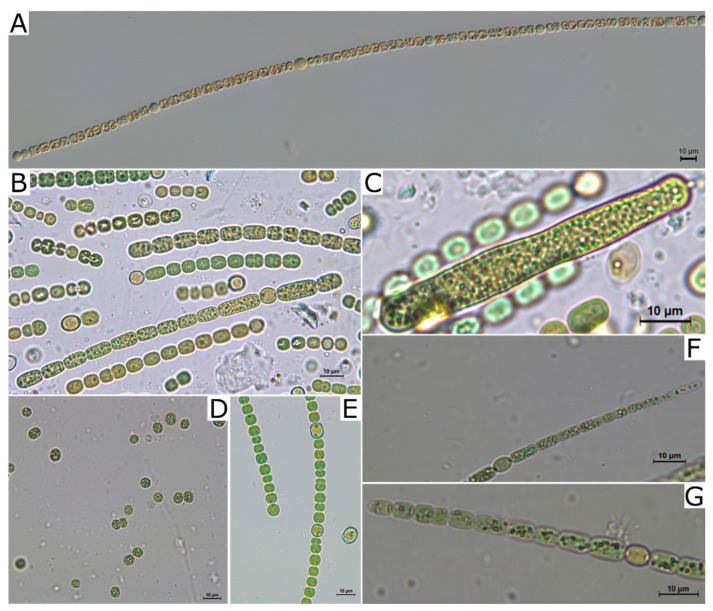
Cyanotoxin-producing strains included in the phylogenetic analysis: BACA0025 (**A–C**), *Microcystis aeruginosa* BACA0148 (**D**), BACA0031 (**E**), and *Aphanizomenon gracile* BACA0041 (**F**,**G**). Scale bars –10 µm.

**Figure 2:**
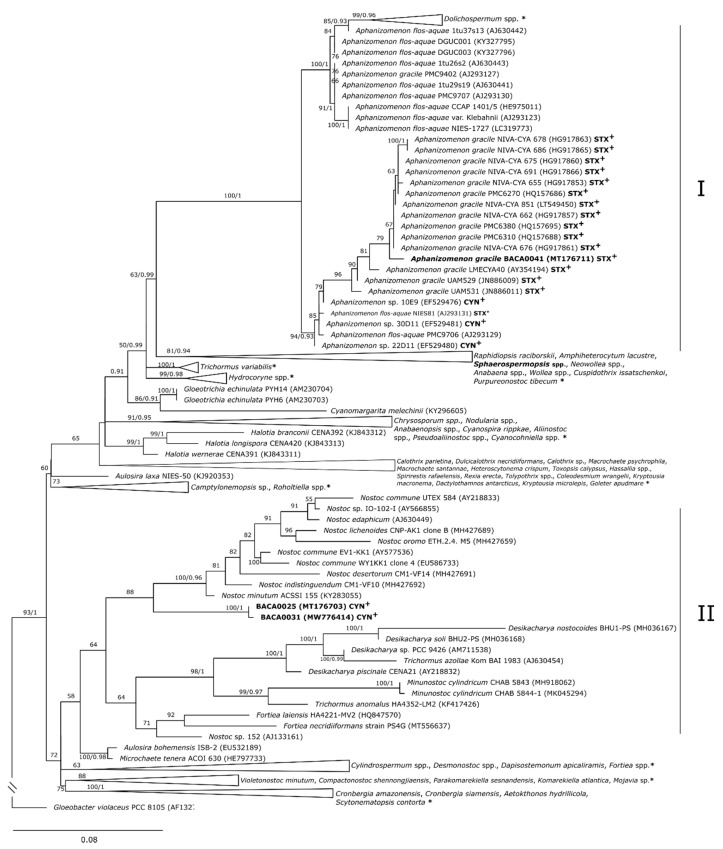
Maximum likelihood tree cluster I of partial 16S rRNA gene sequences (1070 bp). Bootstrap (greater than 50%) and probabilities values (greater than 0.9) are presented in front of the pertinent nodes. **CYN^+^**: Cylindrospermopsin producing strains; STX+: Saxitoxin producing strains. * Collapsed sequences (NCBI accession numbers): AJ630425, FN691925, FN691926, KC297496, FN691923, FN691907, FM242088, FN691908, AB551474, FN691909, KR154316, KR154314, AB551455, KR154298, FN691922, KR154304, AJ630413, AF092504, HQ407325, LR590627, JQ237770, AY763117, AJ582102, LR590629, LR746263, MG921181, MG921182, HQ730086, KT583658, AY701557, FM161349, FM161350, LC474825, LC474824, KT290381, AJ293110, AJ630428, KT290378, KM019920, KT290326, GU434226, AJ630446, MT294032, MT294033, AY196087, KM245026, MN381942, MN381943, DQ234830, AJ630457, AJ630456, KC346266, KC346265, KC346267, JN385287, AF160256, JF768744, EU076457, FJ234895, EF529489, EF529482, EF529488, EU076457, FJ234884, FJ234885, FJ234890, FJ234897, AB608023, JQ237772, LN997860, AJ133177, AJ781131, AJ781149, AJ781145, KM199731, FM177481, AY038033, AM773306, FR774773, MK503791, KY403996, MK503792, MK503793, MH497064, KJ737427, MN243143, AF334695, KY863521, AP018280, KT336439.2, KT336441, MG970541, MG970549, MG970536, MG970538, JN695681, JN695682, FR822753, AF334690, AF334692, JQ083655, KF017617, KF934181, JX088105, HG970655, AF334701, AF334703, KY508609, KY508610, HG970652, MN626663, MN626664, KM199732, KY508607, KY508612, KY508611, KY508608, HG970653, KF417425, JN385289, JN385292, HQ847564, KM268886, KM268884, KY098849, KM268889, KF934180, KF052614, 125687, KF052599, 114701, GQ287650, KF052617, KF052610, KF052603, KF052605, KU161661, AM711524, HG004586, KT166436, MH497066, MH291266, MF642332, KX787933, KJ566945, KJ566947, KF052607, 125685, KF052616, HE797731, KB235930, MN400069, MN400070, MH598843, MT044191, MT044192, KX638483, KX638487, KX638489, KU161674, KU161676, KU161650, AY577534, MF002129, KM019950, AY785313, HQ847558, HQ847561.

The strain BACA0041, morphologically identified as *Aphanizomenon gracile* (Figure 1F,G), was positioned near other *A. gracile* and *Aphanizomenon flos-aquae* strains, and closer to know STX producing strains as *A. gracile* PMC638.10 or *A. flos-aquae* NIES 81 (Figure 2).

Strains BACA0025 (Figure 1A–C) and BACA0031 (Figure 1E), morphologically identified as *Sphaerospermopsis* sp. are positioned in cluster II between *Nostoc* spp., *Fortiea* spp., *Desikacharya* spp., *Trichormus* spp., *Minunostoc cylindricum*, and *Desmonostoc* spp. strains, however, with significant phylogenetic distance from all these strains (Figure 2). 

**Figure 3:**
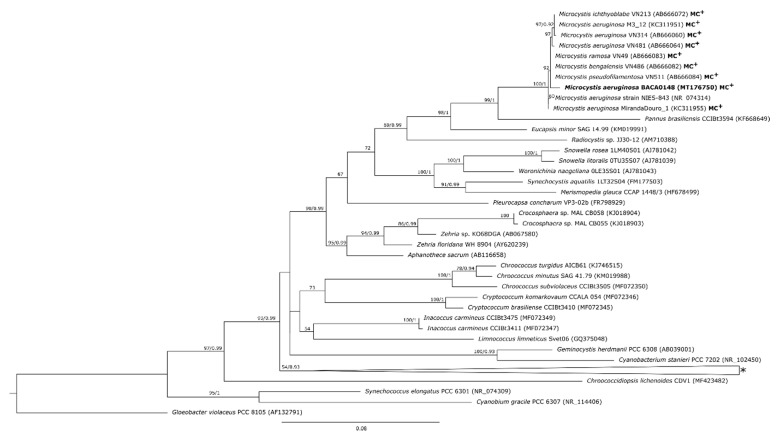
Maximum likelihood tree of the partial 16S rRNA gene sequences (1324 bp). Bootstrap (greater than 50%) and probabilities values (greater than 0.9) are presented in front of the pertinent nodes. MC^+^: Microcystin-producing strains. * Collapsed sequences (NCBI accession numbers): *Myxosarcina* sp. (AJ344562; AJ344561), *Dermocarpella incrassata* (AJ344559), *Synechocystis pevalekii* (KM350249), *Gloeocapsopsis crepidinum* (KF498710), *Foliisarcina bertiogensis* (KT731151), *Hyella patelloides* (HQ832901), *Chroococcidiopsis* sp. (AJ344557), *Xenococcus* sp. (AF132783), *Stanieria cyanosphaera* (112109), *Chroococcopsis gigantea* (KM019987).

The strain BACA0148, morphologically identified as *Microcystis aeruginosa* (Figure 1D), was positioned near other *M. aeruginosa* strains, and closer to know MC producing strains as *M. aeruginosa* VN481 (AB666064) or *M*. *bengalensis* VN486 (AB666082) (Figure 3).

## 3. Discussion

The ability to produce cyanotoxins depends on the simultaneous existence of several genes involved in their biosynthesis pathways [42]. As seen in previous studies, the *sxt* cluster has suffered several modifications and its gene composition and organization varies between taxa [6,43,44,45]. Although the amplification of the *sxt*A gene and the absence of toxin production in *Aphanizomenon* strains was seen in other studies [10,25,46,47], in the studied strains only strains with *sxt*A amplification were able to produce STX (Table 1). Cirés et al. [47] states that the *sxt*A gene does not allow the distinction between STX-producing and non-producing *Anabaena*/*Aphanizomenon* strains, nonetheless the presence of *sxt*A in non-producing strains could have been due to gene loss/inactivation within the *sxt* cluster [46]. The presence of *sxt*G or *sxt*H amplifications in several non-STX producing genera (Table 1; Table S2; Figure S3) in this study are interesting new results that require further investigation to fully understand *sxt* gene distribution among cyanobacteria taxa. Nonetheless, to our knowledge, the presence of *sxt*G, and/or *sxt*H genes in *Kamptonema*, *Leptodesmis*, *Anathece minutissima*, or *Leptolyngbya* strains, have not been reported before.

*Aphanizomenon gracile* BACA0041 was confirmed as an STX producer with amplification of the *sxt*A, *sxt*G, *sxt*H, and *sxt*I genes. The ESI-LC-MS/MS spectra of *A*. *gracile* BACA0041 matched the fragmented pattern of STX standard spectra (Figure S4), with identification of the precursor ion 300 m/z and product ions 282 m/z, 265 m/z, 241 m/z, 240.25 m/z, 204 m/z, and 186 m/z [10]. Phylogenetic analysis confirms *Aphanizomenon gracile* BACA0041 identification’s, this strain is positioned in a well-supported clade of several STX-producing *A. gracile* strains (Figure 2), as *A. gracile* UAM531 [47] and *A. gracile* PMC 638.10 [10]. In our study, we report another freshwater STX-producing *A. gracile* strain, the first STX producer identified in the Azores islands.

Strains BACA0025 and BACA0031 are quite similar, these were both initially identified as *Sphaerospermopsis* sp. (isolated from similar freshwater lakes from the same island; Table S1), with similar BlastN results (Table S3). Strains BACA0025 and BACA0031 were positioned in cluster II in the phylogenetic tree (Figure 2) close to Nostocaceae genera, however with significant phylogenetic distance to conclude that these strains might belong to a new genus. Both were confirmed as CYN producers with detection of both *cyr*B and *cyr*C genes (Table 1). The ESI-LC-MS/MS spectra of BACA0025 and BACA0031 both matched the fragmented pattern of CYN standard spectra (Figure S5), with identification of the precursor ion 416 m/z and product ions 336 m/z, 318 m/z, 274 m/z, and 194 m/z [48], confirming these two strains as CYN producers.

The *cyr*B gene was also amplified in *Nostoc* sp. BACA0109, and in two thermal *Leptolyngbya* sp. strains BACA0142 and BACA0146, however without CYN identification in the ESI-LC-MS/MS. Amplification of *cyr* genes without CYN production confirmation has been reported previously, as is the case of non-CYN producing *Chrysosporum bergii* and *Chrysosporum ovalisporum* strains with amplification of *cyr*A, *cyr*B, and *cyr*C genes [49]. *Nostoc* and *Leptolyngbya* strains are known to produce cyanotoxins, however, MCs [12,17,50] and not CYN, and as far as we know, the presence of the *cyr*B and *cyr*C genes has not been previously reported in *Nostoc* or *Leptolyngbya* strains.

Microcystins are the most common and more well-studied cyanotoxins, being MC-LR one of the most prevalent and toxic congeners [51]. *Microcystis aeruginosa* was the first cyanobacteria species identified as MCs producer and is the most studied species regarding MCs [11]. Our results show that the Azorean strain *M*. *aeruginosa* BACA0148 is also an MC-LR producer, with the detection of *mcy*C, *mcy*D, *mcy*E, and *mcy*G genes. Characteristic MC-LR fragmentation pattern was observed in *M*. *aeruginosa* BACA0148 (Figure S6), with identification of precursor ion 995 m/z and product ions 977 m/z, 866 m/z, 599 m/z, and 553 m/z [52]. The *mcy*E gene amplification in *Nostoc* sp. BACA0091, despite the absence of MC-LR ions in the ESI-LC-MS/MS, or the absence of the other searched *mcy* genes (*mcy*C, *mcy*D, and *mcy*G), can be explained due to gene(s) recombination or loss [6,14,53]. As stated by Dittmann et al. [6], the *mcy* gene cluster has high repetitive sequences, enabling recombination events that ultimately cause changes in the final product, which can be confirmed by the growing reported number of MCs congeners [54,55].

All strains identified as toxin producers (BACA0025, BACA0031, *A. gracile* BACA0041, and *M*. *aeruginosa* BACA0148) were isolated from lakes in Pico and São Miguel islands (Table S1, S2). The presence of toxic strains in these lakes represents environmental and public health hazards. Contrarily, none of the strains isolated from thermal and terrestrial habitats were identified as cyanotoxin producers, although in some of them, cyanotoxin-encoding genes were detected, as in the thermal *Leptolyngbya* strains BACA0112, BACA0123, BACA0142, BACA0144 and BACA0146, and *Coleospermum* sp. BACA0119.

## 4. Conclusions

Within the BACA collection, we identified and reported another MC-LR-producing *M*. *aeruginosa* strain (BACA0148) and another STX-producing *A*. *gracile* strain (BACA0041). Phylogenetic analysis revealed evidence for new cyanobacteria taxa BACA0025 and BACA0031, confirmed as CYN producers. Further studies are necessary to confirm and describe these new taxa, with morphological characterization and 16S rRNA and ITS analysis.

The identification of new cyanotoxin-producing strains, and unreported toxins, in the Azores, confirms the risk of toxicity and threat to environmental and public health; thus, an appropriate monitoring program should be implemented/updated to search MCs, STX, and CYN. Future efforts should also be made to avoid cyanobacteria blooms and consequently cyanotoxins released in high concentrations. 

## 5. Materials and Methods

### 5.1. BACA Strains and Growth Conditions 

A total of 157 strains, isolated from various environments (Table S1), were retrieved from the Azorean Bank of Algae and Cyanobacteria (BACA) created in the framework of the REBECA project (MAC/1.1a/060). For genetic analysis, 50 mL cultures were prepared without agitation, whereas for toxin extraction, the cultures were scaled up to 1 L, with filtered aeration. All strains were grown in liquid BG-11 media (with or without combined nitrogen) [56], in a climate-controlled room with a 14:10 h light: dark (170 µmol photons m^−2^ s^−1^) photoperiod at 25 °C [56,57]. Cyanobacterial cells were harvested by centrifugation (4000× *g* for 15 min), after 3–5 weeks, and lyophilized. The lyophilized cyanobacteria biomass were stored at −20 °C.

### 5.2. Cyanotoxins Analysis

#### 5.2.1. Toxin extraction

The lyophilized cyanobacteria, 157 samples in total, were weighed (80–100 mg) to a glass vial and extracted with a 5% methanolic solution (2–10 mL). Solutions were then submitted to ultrasounds for 1–5 min, 60 Hz, in an ice bath, and transferred to falcons to be centrifuged (5000× *g*, 5 min, 4°C). Pellet was then submitted to a second extraction and left in the dark at 4 °C overnight. Supernatants were pooled together and lyophilized (extraction solvent completely freeze-dried).

Residues were finally dissolved in 200–500 µL 50% methanol LC-MS grade acidified with 0.1% Formic Acid and filtered with a nylon membrane 0.2 µm before analysis (or centrifuged at 10,000× *g* for 5 min). Samples were protected from light through all the processes and stored at −80 °C until analysis.

#### 5.2.2. ESI-LC-MS/MS analysis 

Saxitoxin (CRM-00-STX, Lot 16-001, 99% purity), microcystin-LR (CRM-00-MC-LR, Lot 19-001, 96% purity), and cylindrospermopsin (CRM-03-CYN, Lot 16-001, 99% purity) standards were all supplied by Cifga (Lugo, Spain). Although MC-RR, MC-YR, and MC-LA standards were not used, their mass was searched in the spectra.

All the standards were injected individually and then as a standard mixture with a concentration interval from 10 ppb to 300 ppb. ESI-LC-MS/MS analysis was performed to confirm the presence or absence of these three cyanotoxins in the selected cyanobacteria strains. 

Samples were injected in a Liquid Chromatograph Thermo Finnigan Surveyor HPLC System (Thermo Scientific, Waltham, MA, USA), coupled with a Mass Spectrometry LCQ Fleet™ Ion Trap Mass Spectrometer (Thermo Scientific, Waltham, MA, USA), with a column TSKgel^®^ Amide-80 Phase carbamoyl (250 mm× 2 mm i.d., 5 µm) (TOSOH Bioscience-Lot082B, Tokyo, Japan). 

The eluents used were methanol (A) and water (B) both acidified with formic acid at 0.1% (v/v). The gradient program started at 10% B (held for 10 min), increasing to 50% B in 5 min, turning back to initial conditions in 5 min, equilibrating more 10 min with 10% B. The injection volume was 10 µL with a flow of 0.2 mL min^−1^ and column kept at 30 °C. 

Mass spectrometry analysis acquisition parameters were as follows: ESI source, positive ionization using collision-induced dissociation (CID). Table S4 resumes analysis parameters for each searched toxin.

### 5.3. DNA Extraction, PCR Amplification, and Sequencing

Total genomic DNA was extracted with the PureLinkTM Genomic DNA Mini Kit (Invitrogen, Carlsbad, CA, USA), as previously described by Cordeiro et al. [58]. DNA samples were stored at −20 °C.

Genes *mcy*C, *mcy*D, *mcy*E, and *mcy*G were targeted for MCs production potential, *sxt*A, *sxt*G, *sxt*I, and *sxt*H for STX, and *cyr*B and *cyr*C for CYN, using specific primer pairs available in the literature (Table 2). For the 16S rRNA gene amplification primers 27F [59], CYA359F [60], and 1494R [59] were used, whereas for sequencing it was also used primers CYA781F [60] and CYA781R (Table 2).

PCRs were carried out in a ProFlex™ 3 × 32-well PCR System (Thermo Fischer, Waltham, MA, USA), according to the literature [9,10,25,58,61,62]. The PCR products were visualized by electrophoresis on 1.5% agarose gels stained with SYBR™ SAFE (0.2 g mL^−1^) and visualized using the transilluminator Molecular Imager^®^ Gel Doc™ XR^+^ (BioRad, Hercules, CA, USA).

16S rRNA amplification products were purified using the EXTRACTME^®^ DNA clean-up kit (Blirt, Gdańsk, Poland), following the manufacturer’s protocol. Sequencing was done by Macrogen Ltd. (Madrid, Spain). Nucleotide sequences were deposited in the NCBI Genbank under the accession numbers MT176703, MW776414, MT176711, and MT176750.

### 5.4. Phylogenetic Analysis

Partial 16S sequences were amplified for the four strains with toxin identification by ESI-LC-MS/MS. All the sequences obtained in this study were compared with sequences deposited in the GenBank NCBI by BlastN tool.

The databases were constructed with the 16S rRNA sequences from this study and identified strains with MCs, STX, and CYN production retrieved from the literature [10,17,25,43,63,64,65,66,67] and GenBank. A final database of 267 OTUs (operational taxonomic units) were aligned for Nostocales and 50 OTUs for Chroococcales, using MAFFT v7.475 [68]. The sequence data matrixes with a final length of 1070 bp (Nostocales) and 1324 bp (Chroococcales) were used to infer phylogenetic distances.

The 16S rRNA gene phylogenetic relations were calculated using maximum likelihood (ML) and Bayesian inference (BI). jModelTest 2.1.10 [69] was used to select the best-fit nucleotide model for our database, on which the general time-reversible evolutionary model of substitution with gamma-distributed evolutionary rates and with an estimated proportion of invariable sites (GTR+G+I) was selected. ML was calculated using the IQ-Tree online version v1.6.12 [70] with 1000 ultrafast bootstrap and BI was calculated using MrBayes v3.2.7a [71], applying two separate runs with four chains each and 50,000,000 Markov chain Monte Carlo generations (sampling every 100 generations with a 0.25 burn-in). The tree was drawn with FigTree 1.4.4 (http://tree.bio.ed.ac.uk/software/figtree, accessed on 8 February 2021) and Inkscape 1.0.1 (https://inkscape.org/pt/, accessed on 8 February 2021). Only the ML tree is presented, with bootstrap percentages (ML) and BI probabilities for branch support, since ML and BI methods resulted in similar trees. Only probabilities above 0.9 and bootstrap percentages above 50 are shown at the branch nodes of the phylogenetic distance trees. *Gloeobacter violaceus* PCC 8105 (AF132791) was used as the out-group.

## Figures and Tables

**Table 1:** Detection of genes involved in microcystin, cylindrospermopsin, and saxitoxin production, and ESI-LC-MS/MS analysis on BACA strains. Only strains with positive detection of either the genes or the toxins are shown.

Strains	Habitat	Species	PCR			ESI-LC-MS/MS		
			SXT	CYN	MC	SXT	CYN	MC-LR
BACA0007	Lake	*Kamptonema* sp.	G	-	-	-	-	-
BACA0025	Lake	unidentified species	-	B, CC	-	-	+	-
BACA0031	Lake	unidentified species	-	B, CC	-	-	+	-
BACA0041	Lake	*Aphanizomenon gracile*	A, G, H, I	-	-	+	-	-
BACA0081	Lake	*Cylindrospermum* sp.	H	-	-	-	-	-
BACA0091	Lake	*Nostoc* sp.	-	-	E	-	-	-
BACA0109	Lake	*Nostoc* sp.	-	B	-	-	-	-
BACA0142	Thermal	*Leptolyngbya* sp.	-	B	-	-	-	-
BACA0146	Thermal	*Leptolyngbya* sp.	-	B	-	-	-	-
BACA0148	Lake	*Microcystis aeruginosa*	-	-	C, D, E, G	-	-	+
BACA0203	Lake	*Leptodesmis* sp.	G, H	-	-	-	-	-
BACA0204	Lake	*Leptolyngbya* sp.	G	-	-	-	-	-
BACA0223	Lake	*Anathece minutissima*	G	-	-	-	-	-

“-“: absence of biosynthesis-encoding genes amplification or absence of toxin in the ESI-LC-MS/MS analysis; C: *mcy*C, D: *mcy*D, E: *mcy*E, G: *mcy*G, A: *sxt*A, G: *sxt*G, H: *sxt*H, I: *sxt*I, B: *cyr*B, CC: *cyr*C, amplification of respective biosynthesis-encoding genes.

**Table 2:** Primers used to amplify and/ or sequence cyanotoxins biosynthesis genes and 16S rRNA.

Gene	Primer	Fragment length (bp)	Sequence (5’–3’)	References
*mcy*C	PSCF1	674	GCAACATCCCAAGAGCAAAG	Ouahid et al. [61]
	PSCR1		CCGACAACATCACAAAGGC	
*mcy*D	PKDF1	647	GACGCTCAAATGATGAAAC	
	PKDR1		GCAACCGATAAAAACTCCC	
*mcy*E	PKEF1	755	CGCAAACCCGATTTACAG	
	PKER1		CCCCTACCATCTTCATCTTC	
*mcy*G	PKGF1	425	ACTCTCAAGTTATCCTCCCTC	
	PKGR1		AATCGCTAAAACGCCACC	
*sxt*A	sxtA F	602	AGGTCTTTGACTTGCATCCAA	Ledreux et al. [10]
	sxtA R		AACCGGCGACATAGATGATA	
*sxt*G	sxtGf	893	AGGAATTCCCTATCCACCGGAG	Casero et al. [25]
	sxtGr		CGGCGAACATCTAACGTTGCAC	
*sxt*H	sxtHf	812	AAGACCACTGTCCCCACCGAGG	
	sxtHr		CTGTGCAGCGATCTGATGGCAC	
*sxt*I	sxtIf	910	AGCGCTGCCGCTATGGTTGTCG	
	sxtIr		ACGCAATTGAGGGCGACACCAC	
*cyrB*	M13	597	GGCAAATTGTGATAGCCACGAGC	Schembri et al. [62]
	M14		GATGGAACATCGCTCACTGGTG	
*cyr*C	M4	650	GAAGCTCTGGAATCCGGTAA	
	M5		AATCCTTACGGGATCCGGTGC	
16S rRNA	27F		AGAGTTTGATCCTGGCTCAG	Neilan et al. [59]
	CYA359F		GGGGAATYTTCCGCAATG GG	Nubel et al. [60]
	1494R		TACGGCTACCTTGTTACGAC	Neilan et al. [59]
	CYA781R		GACTACTGGGGTATCTAATCCCATT	Nubel et al. [60]
	CYA781F		AATGGGATTAGATACCCCAGTAGTC	This study

## Data Availability

Data is contained within the article or Supplementary Material. The data presented in this study are available in https://www.mdpi.com/article/10.3390/toxins13040258/s1.

## Supplementary Materials

The following are available online at https://www.mdpi.com/article/10.3390/toxins13040258/s1, Figure S1: Electrophoresis gel photos of *mcy*C (674 bp), *mcy*D (647 bp), *mcy*E (755 bp), and *mcy*G (425 bp) biosynthesis genes amplifications, Figure S2: Electrophoresis gel photos of *cyr*B (650 bp) and *cyr*C (597 bp) biosynthesis genes amplifications, Figure S3: Electrophoresis gel photos of *sxt*A (602 bp), *sxt*G (893 bp), *sxt*H (812 bp), and *sxt*I (910 bp) biosynthesis genes amplifications, Figure S4: Total ion chromatograms and spectra of a STX standard solution (**A**) and sample *Aphanizomenon gracile* BACA0041 (**B**), Figure S5: Total ion chromatograms and spectra of a CYN standard solution (**A**), sample BACA0025 (**B**) and sample BACA0031 (**C**), Figure S6. Total ion chromatograms and spectra of an MC-LR standard solution (**A**) and sample *Microcystis aeruginosa* BACA0148 (**B**), Table S1: Strains information, Table S2: PCR amplifications of MC (*mcy*C, *mcy*D, *mcy*E, *mcy*G), STX (*sxt*A, *sxt*G, *sxt*H, *sxt*I) and CYN (*cyr*B, *cyr*C) biosynthesis-encoding genes and ESI-LC-MS/MS toxicity confirmation, Table S3: Sequence identity (%) of 16S rRNA gene fragment between BACA strains and other cyanobacterial sequences available in GenBank (NCBI), Table S4: ESI-LC-MS/MS analysis parameters for the identification of CYN, MC-LR, and STX.
