# Supplementary material for: Cyanotoxin Screening in BACA Culture Collection: Identification of New Cylindrospermopsin Producing Cyanobacteria
Source: Toxins (Basel). 2021 Apr 3;13(4):258. doi: 10.3390/toxins13040258 (PMC8065757; doi:10.3390/toxins13040258)
Supplement: Supplementary file 1 [file toxins-13-00258-s001.zip › Supplementary_Tables_2.pdf]

Table S1. BACA strains information, taxonomical identification and habitat.

Strain Code	Order	Family	Species	Ecosystem	Habitat	Local	Island
BACA0001	*Nostocales*	*Nostocaceae*	*Nostoc* sp.	Freshwater Lake	Plankton	Lagoa do Peixinho	Pico
BACA0002	*Nostocales*	*Nostocaceae*	*Nostoc* sp.	Freshwater Lake	Plankton	Lagoa das Furnas	São Miguel
BACA0003	*Nostocales*	*Nostocaceae*	*Trichormus* sp.	Freshwater Lake	Plankton	Lagoa do Capitão	Pico
BACA0004	*Nostocales*	*Rivulariaceae*	*Rivularia* sp.	Freshwater Lake	Plankton	Lagoa Azul	São Miguel
BACA0005	*Nostocales*	*Scytonemataceae*	*Scytonematopsis* sp.	Freshwater Lake	Plankton	Lagoa do Peixinho	Pico
BACA0006	*Nostocales*	*Rivulariaceae*	*Calothrix* sp.	Freshwater Lake	Plankton	Lagoa das Empadadas Norte	São Miguel
BACA0007	*Oscillatoriales*	*Oscillatoriaceae*	*Kamptonema* sp.	Freshwater Lake	Plankton	Lagoas das Empadadas Sul	São Miguel
BACA0008	*Oscillatoriales*	*Microcoleaceae*	*Arthrospira jenneri*	Freshwater Lake	Plankton	Lagoa do Pico D'el Rei	São Miguel
BACA0009	*Nostocales*	*Nostocaceae*	*Nostoc paludosum*	Freshwater Lake	Plankton	Lagoa Azul	São Miguel
BACA0010	*Nostocales*	*Nostocaceae*	*Nostoc punctiforme*	Freshwater Lake	Plankton	Lagoa de São Brás	São Miguel
BACA0011	*Oscillatoriales*	*Oscillatoriaceae*	*Arthrospira jenneri*	Freshwater Lake	Plankton	Lagoa do Areeiro	São Miguel
BACA0012	*Synechococcales*	*Oculatellaceae*	Tildeniella *sp.*	Freshwater Lake	Plankton	Lagoa das Furnas	São Miguel
BACA0013	*Nostocales*	*Nostocaceae*	*Nostocaceae*	Freshwater Lake	Plankton	Lagoa Azul	São Miguel
BACA0014	*Synechococcales*	*Pseudanabaenaceae*	*Pseudanabaena minima*	Freshwater Lake	Plankton	Lagoa Rasa da Serra Devassa	São Miguel
BACA0015	*Synechococcales*	*Pseudanabaenaceae*	*Pseudanabaena* sp.	Freshwater Lake	Plankton	Lagoa do Areeiro	São Miguel
BACA0016	*Synechococcales*	*Pseudanabaenaceae*	*Pseudanabaena limnetica*	Freshwater Lake	Plankton	Lagoa do Caiado	Pico
BACA0017	*Nostocales*	*Microchaetaceae*	*Microchaete tenera*	Freshwater Lake	Plankton	Lagoa Rosada	Pico
BACA0018	*Nostocales*	*Microchaetaceae*	*Coleospermum* sp.	Freshwater Lake	Plankton	Lagoa Comprida	Flores
BACA0019	*Synechococcales*	*Synechococcaceae*	*Cyanobium sp.*	Freshwater Lake	Plankton	Lagoa Azul	São Miguel
BACA0020	*Nostocales*	*Scytonemataceae*	*Scytonematopsis* sp.	Freshwater Lake	Plankton	Lagoa Comprida	Flores
BACA0021	*Nostocales*	*Microchaetaceae*	*Coleospermum* sp.	Freshwater Lake	Plankton	Lagoa do Peixinho	Pico
BACA0022	*Nostocales*	*Fortieaceae*	*Fortiea* sp.	Freshwater Lake	Plankton	Lagoa do Paul	Pico
BACA0023	*Nostocales*	*Nostocaceae*	*Nostoc commune*	Freshwater Lake	Plankton	Lagoa do Peixinho	Pico
BACA0024	*Synechococcales*	*Leptolyngbyaceae*	*Planktolyngbya* sp.	Freshwater Lake	Plankton	Lagoa Azul	São Miguel
BACA0025	*Nostocales*	*Aphanizomenonaceae*	*unidentified species*	Freshwater Lake	Plankton	Lagoa Rosada	Pico
BACA0026	*Nostocales*	*Nostocaceae*	*Nostoc punctiforme*	Freshwater Lake	Plankton	Lagoa de São Brás	São Miguel
BACA0027	*Synechococcales*	*Pseudanabaenaceae*	*Limnothrix* sp.	Freshwater Lake	Plankton	Lagoa das Furnas	São Miguel
BACA0028	*Nostocales*	*Tolypothrichaceae*	*Tolypothrix* s.l.	Freshwater Lake	Plankton	Lagoa Rasa das Sete Cidades	São Miguel
BACA0029	*Nostocales*	*Aphanizomenonaceae*	*Aphanizomenon* sp.	Freshwater Lake	Plankton	Lagoa Azul	São Miguel
BACA0030	*Nostocales*	*Aphanizomenonaceae*	*Dolichospermum* sp.	Freshwater Lake	Plankton	Lagoa Azul	São Miguel

BACA0031	*Nostocales*	*Aphanizomenonaceae*	*unidentified species*	Freshwater Lake	Plankton	Lagoa do Caiado	Pico
BACA0032	*Nostocales*	Scytonemataceae	*Scytonematopsis sp.*	Freshwater Lake	Plankton	Lagoa do Congro	São Miguel
BACA0033	*Nostocales*	*Microchaetaceae*	*Coleospermum* sp.	Freshwater Lake	Plankton	Lagoa Comprida	Flores
BACA0034	*Nostocales*	*Aphanizomenonaceae*	*Dolichospermum* sp.	Freshwater Lake	Plankton	Lagoa do Fogo	São Miguel
BACA0035	*Nostocales*	*Nostocaceae*	*Nostoc* sp.	Freshwater Lake	Plankton	Lagoa das Furnas	São Miguel
BACA0036	*Nostocales*	*Nostocaceae*	*Nostoc* sp.	Freshwater Lake	Plankton	Lagoa da Lomba	Flores
BACA0037	*Nostocales*	*Microchaetaceae*	*Coleospermum* sp.	Freshwater Lake	Plankton	Lagoa da Lomba	Flores
BACA0038	*Nostocales*	*Nostocaceae*	cf. *Isocystis planctonica*	Freshwater Lake	Plankton	Lagoa da Lomba	Flores
BACA0039	*Nostocales*	*Nostocaceae*	*Nostoc* sp.	Freshwater Lake	Plankton	Lagoa Negra	Flores
BACA0040	*Nostocales*	*Microchaetaceae*	*Coleospermum* sp.	Freshwater Lake	Plankton	Lagoa do Peixinho	Pico
BACA0041	*Nostocales*	*Aphanizomenonaceae*	*Aphanizomenon gracile*	Freshwater Lake	Plankton	Lagoa de Santiago	São Miguel
BACA0042	*Nostocales*	*Nostocaceae*	*Nostoc* sp.	Freshwater Lake	Plankton	Lagoa de Santiago	São Miguel
BACA0043	*Nostocales*	*Tolypothrichaceae*	*Tolypothrix sp.*	Freshwater Lake	Plankton	Lagoa Verde	São Miguel
BACA0044	*Nostocales*	*Aphanizomenonaceae*	*Dolichospermum delicatulum*	Freshwater Lake	Plankton	Lagoa do Fogo	São Miguel
BACA0045	*Synechococcales*	*Pseudanabaenaceae*	*Limnothrix* sp.	Freshwater Lake	Plankton	Lagoa das Furnas	São Miguel
BACA0046	*Nostocales*	*Microchaetaceae*	*Coleospermum* sp.	Freshwater Lake	Plankton	Lagoa da Lomba	Flores
BACA0047	*Nostocales*	*Rivulariaceae*	*Calothrix* sp.	Freshwater Lake	Plankton	Lagoa Funda	Flores
BACA0048	*Synechococcales*	*Pseudanabaenaceae*	*Limnothrix* sp.	Freshwater Lake	Plankton	Lagoa Funda	Flores
BACA0049	*Nostocales*	*Tolypothrichaceae*	*Tolypothrix* sp.	Freshwater Lake	Plankton	Lagoa do Capitão	Pico
BACA0050	*Nostocales*	*Aphanizomenonaceae*	*Dolichospermum* s.l.	Freshwater Lake	Plankton	Lagoa Rosada	Pico
BACA0051	*Nostocales*	*Nostocaceae*	*Nostoc* sp.	Freshwater Lake	Plankton	Lagoa do Caldeirão	Corvo
BACA0052	*Nostocales*	*Nostocaceae*	*Nostoc paludosum*	Freshwater Lake	Plankton	Lagoa do Caldeirão	Corvo
BACA0053	*Nostocales*	*Nostocaceae*	*Nostoc* cf. *sphaericum*	Freshwater Lake	Plankton	Lagoa do Caldeirão	Corvo
BACA0054	*Synechococcales*	*Leptolyngbyaceae*	*Stenomitos* sp.	Freshwater Lake	Plankton	Lagoa do Peixinho	Pico
BACA0055	*Nostocales*	*Microchaetaceae*	*Microchaete tenera*	Freshwater Lake	Plankton	Lagoa Rosada	Pico
BACA0056	*Nostocales*	*Tolypothrichaceae*	*Tolypothrix* cf. *helicophila*	Freshwater Lake	Plankton	Lagoa Rosada	Pico
BACA0057	*Nostocales*	*Nostocaceae*	*Nostoc* sp.	Freshwater Lake	Plankton	Lagoa do Canário	São Miguel
BACA0058	*Nostocales*	*Nostocaceae*	*Nostoc* sp.	Freshwater Lake	Plankton	Lagoa do Fogo	São Miguel
BACA0059	*Nostocales*	*Nostocaceae*	*Nostoc* sp.	Freshwater Lake	Plankton	Lagoa das Furnas	São Miguel
BACA0060	*Nostocales*	*Rivulariaceae*	*Calothrix* sp.	Freshwater Lake	Plankton	Lagoa das Furnas	São Miguel
BACA0061	*Nostocales*	*Nostocaceae*	*Nostoc* sp.	Freshwater Lake	Plankton	Lagoa da Lomba	Pico
BACA0062	*Nostocales*	*Microchaetaceae*	*Microchaete tenera*	Freshwater Lake	Plankton	Lagoa da Lomba	Pico
BACA0063	*Nostocales*	*Nostocaceae*	*Nostoc* sp.	Freshwater Lake	Plankton	Lagoa de São Brás	São Miguel

BACA0064	*Nostocales*	*Tolypothrichaceae*	*Tolypothrix* sp.	Freshwater Lake	Plankton	Lagoa do Paul	Pico
BACA0065	*Cyanobacterium*	*Cyanobacteriaceae*	*Cyanobacterium* cf. *synechococcoides*	Freshwater Lake	Plankton	Lagoa das Furnas	São Miguel
BACA0066	*Nostocales*	*Tolypothrichaceae*	*Tolypothrix* sp.	Freshwater Lake	Plankton	Lagoa do Congro	São Miguel
BACA0067	*Nostocales*	*Nostocaceae*	*Anabaena* sp.	Freshwater Lake	Plankton	Lagoas das Empadadas Sul	São Miguel
BACA0068	*Nostocales*	*Nostocaceae*	*Nostoc* sp.	Freshwater Lake	Plankton	Lagoa do Canário	São Miguel
BACA0069	*Nostocales*	*Nostocaceae*	*Nostoc* sp.	Freshwater Lake	Plankton	Lagoa do Congro	São Miguel
BACA0070	*Nostocales*	*Rivulariaceae*	*Calothrix* sp.	Freshwater Lake	Plankton	Lagoa do Congro	São Miguel
BACA0071	*Synechococcales*	*Leptolyngbyaceae*	*Planktolyngbya* cf. *limnetica*	Freshwater Lake	Plankton	Lagoa Negra	Flores
BACA0072	*Nostocales*	*Rivulariaceae*	*Calothrix* sp.	Freshwater Lake	Plankton	Lagoa Verde	São Miguel
BACA0073	*Nostocales*	*Rivulariaceae*	*Rivularia* sp.	Freshwater Lake	Plankton	Lagoa de Santiago	São Miguel
BACA0074	*Nostocales*	*Tolypothrichaceae*	*Tolypothrix* sp.	Freshwater Lake	Plankton	Lagoa do Fogo	São Miguel
BACA0075	*Chroococcales*	*Aphanothecaceae*	*Aphanothece* sp.	Freshwater Lake	Plankton	Lagoa das Furnas	São Miguel
BACA0076	*Synechococcales*	*Synechococcaceae*	*Cyanobium* cf. *planctum*	Freshwater Lake	Rock biofilm	Lagoa Sete Cidades	São Miguel
BACA0077	*Synechococcales*	*Oculatellaceae*	*Pegethrix* sp.	Freshwater Lake	Rock biofilm	Lagoa Sete Cidades	São Miguel
BACA0078	*Oscillatoriales*	*Oscillatoriaceae*	*Phormidium* sp.	Freshwater Lake	Plankton	Lagoa do Capitão	Pico
BACA0079	*Nostocales*	*Nostocaceae*	*Nostoc* s.l.	Freshwater Lake	Plankton	Lagoa do Capitão	Pico
BACA0080	*Nostocales*	*Rivulariaceae*	*Calothrix* sp.	Freshwater Lake	Plankton	Lagoa das Empadadas Norte	São Miguel
BACA0081	*Nostocales*	*Nostocaceae*	*Cylindrospermum* sp.	Freshwater Lake	Plankton	Lagoa Rasa das Sete Cidades	São Miguel
BACA0082	*Nostocales*	*Nostocaceae*	*Nostoc* sp.	Freshwater Lake	Plankton	Lagoa Negra	Flores
BACA0083	*Nostocales*	*Rivulariaceae*	*Rivularia* sp.	Freshwater Lake	Plankton	Lagoa Verde	São Miguel
BACA0084	*Nostocales*	*Microchaetaceae*	*Coleospermum* sp.	Freshwater Lake	Plankton	Lagoa do Fogo	São Miguel
BACA0085	*Nostocales*	*Microchaetaceae*	*Coleospermum* sp.	Freshwater Lake	Plankton	Lagoa do Caiado	Pico
BACA0086	*Nostocales*	*Nostocaceae*	*Nostoc* sp.	Freshwater Lake	Plankton	Lagoa Rasa	Flores
BACA0087	*Nostocales*	*Nostocaceae*	*Nostoc* sp.	Freshwater Lake	Plankton	Lagoa de Santiago	São Miguel
BACA0090	*Nostocales*	*Hapalosiphonaceae*	*Hapalosiphon* sp.	Terrestrial	Biofilm	Sete Cidades	São Miguel
BACA0091	*Nostocales*	*Nostocaceae*	*Nostoc* sp.	Terrestrial	Biofilm	Sete Cidades	São Miguel
BACA0092	*Nostocales*	*Nostocaceae*	*Nostoc* sp.	Stream	Benthic	Lagoa do Fogo	São Miguel
BACA0093	*Nostocales*	*Tolypothrichaceae*	*Tolypothrix* sp.	Stream	Benthic	Ribeira do Guilherme	São Miguel
BACA0094	*Nostocales*	*Rivulariaceae*	*Calothrix* sp.	Stream	Benthic	Ribeira do Guilherme	São Miguel
BACA0095	*Nostocales*	*Nostocaceae*	*Nostoc* sp.	Tree	Biofilm	Janela do Inferno	São Miguel
BACA0096	*Nostocales*	*Nostocaceae*	*Nostoc* sp.	Tree	Biofilm	Janela do Inferno	São Miguel
BACA0097	*Nostocales*	*Nostocaceae*	*Cylindrospermum cf. muscicola*	Terrestrial	Biofilm	Lagoa do Canário	São Miguel
BACA0098	*Nostocales*	*Tolypothrichaceae*	*Tolypothrix* sp.	Stream	Benthic	Ribeira do Guilherme	São Miguel

BACA0099	*Nostocales*	*Nostocaceae*	*Nostoc* sp.	Terrestrial	Biofilm	Sete Cidades	São Miguel
BACA0100	*Nostocales*	*Rivulariaceae*	*Calothrix* sp.	Stream	Benthic	Ribeira do Salto	Santa Maria
BACA0101	*Nostocales*	*Microchaetaceae*	*Coleospermum* sp.	Terrestrial	Biofilm	Lagoa do Canário (Soil Biofilm)	São Miguel
BACA0102	*Nostocales*	*Microchaetaceae*	*Coleospermum* sp.	Terrestrial	Biofilm	Lagoa do Canário (Soil Biofilm)	São Miguel
BACA0103	*Nostocales*	*Nostocaceae*	*Nostoc* sp.	Terrestrial	Biofilm	Lagoa do Canário (Soil Biofilm)	São Miguel
BACA0104	*Nostocales*	*Nostocaceae*	*Nostoc* sp.	Stream	Benthic	Ribeira de São Francisco	Santa Maria
BACA0105	*Nostocales*	*Rivulariaceae*	*Calothrix* sp.	Stream	Benthic	Ribeira de São Francisco	Santa Maria
BACA0106	*Oscillatoriales*	*Oscillatoriaceae*	*Tychonema* sp.	Artificial lake	Plankton	Furnas	São Miguel
BACA0107	*Nostocales*	*Nostocaceae*	*Hydrocoryne* cf. *spongiosa*	Artificial lake	Plankton	Furnas	São Miguel
BACA0108	*Oscillatoriales*	*Oscillatoriaceae*	*Lyngbya* cf. *martensiana*	Cave	Rock biofilm	Furnas	São Miguel
BACA0109	*Nostocales*	*Nostocaceae*	*Nostoc* sp.	Freshwater Lake	Plankton	Lagoa do Paul	Pico
BACA0110	*Nostocales*	*Microchaetaceae*	*Coleospermum* sp.	Thermal pool	Benthic	Poça da Silvina	São Miguel
BACA0111	*Nostocales*	*Microchaetaceae*	*Microchaete bulbosa*	Thermal spring	Benthic	Água de Prata	São Miguel
BACA0112	*Synechococcales*	*Leptolyngbyaceae*	*Leptolyngbya gelatinosa*	Thermal spring	Benthic	Água do Rego	São Miguel
BACA0113	*Oscillatoriales*	*Oscillatoriaceae*	*Phormidium* sp.	Thermal stream	Benthic	Ribeira Amarela	São Miguel
BACA0114	*Nostocales*	*Hapalosiphonaceae*	*Westiellopsis* sp.	Thermal stream	Benthic	Ribeira Amarela	São Miguel
BACA0116	*Nostocales*	*Chlorogloeopsidaceae*	*Chlorogloeopsis fritschii*	Thermal pool	Benthic	Poça da Silvina	São Miguel
BACA0117	*Nostocales*	*Microchaetaceae*	*Coleospermum* sp.	Thermal stream	Benthic	Ribeira Amarela	São Miguel
BACA0118	*Nostocales*	*Hapalosiphonaceae*	*Mastigocladus* sp.	Thermal stream	Benthic	Ribeira Amarela	São Miguel
BACA0119	*Nostocales*	*Microchaetaceae*	*Coleospermum* sp.	Thermal spring	Benthic	Nascente da Morangueira	São Miguel
BACA0120	*Nostocales*	*Microchaetaceae*	*Coleospermum* sp.	Thermal pool	Benthic	Poça da Silvina	São Miguel
BACA0121	*Nostocales*	*Hapalosiphonaceae*	*Mastigocladus laminosus*	Thermal spring	Benthic	Água do Rego	São Miguel
BACA0122	*Nostocales*	*Hapalosiphonaceae*	*Mastigocladus laminosus*	Thermal pool	Benthic	Poça da Silvina	São Miguel
BACA0123	*Synechococcales*	*Leptolyngbyaceae*	*Leptolyngbya granulifera*	Thermal spring	Benthic	Nascente Água das Quenturas	São Miguel
BACA0124	*Nostocales*	*Microchaetaceae*	*Coleospermum* sp.	Fumarole	Biofilm	Caldeira do Esguicho	São Miguel
BACA0125	*Nostocales*	*Hapalosiphonaceae*	*Mastigocladus laminosus*	Thermal pool	Benthic	Poça da Silvina	São Miguel
BACA0126	*Nostocales*	*Chlorogloeopsidaceae*	*Chlorogloeopsis fritschii*	Thermal pool	Benthic	Poça da Silvina	São Miguel
BACA0127	*Synechococcales*	*Leptolyngbyaceae*	*Leptolyngbya* cf. *subuliformis*	Thermal spring	Benthic	Nascente Água das Quenturas	São Miguel
BACA0128	*Nostocales*	*Hapalosiphonaceae*	*Mastigocladus laminosus*	Thermal spring	Benthic	Nascente Água das Quenturas	São Miguel
BACA0129	*Nostocales*	*Chlorogloeopsidaceae*	*Chlorogloeopsis fritschii*	Thermal spring	Benthic	Nascente Água das Quenturas	São Miguel
BACA0130	*Nostocales*	*Hapalosiphonaceae*	*Mastigocladus laminosus*	Thermal pool	Benthic	Furnas	São Miguel
BACA0131	*Nostocales*	*Chlorogloeopsidaceae*	*Chlorogloeopsis fritschii*	Thermal pool	Benthic	Poça da Silvina	São Miguel

BACA0132	*Nostocales*	*Hapalosiphonaceae*	*Mastigocladus* sp.	Thermal spring	Benthic	Nascente Água das Quenturas	São Miguel
BACA0133	*Nostocales*	*Microchaetaceae*	*Coleospermum* sp.	Thermal spring	Benthic	Nascente Água das Quenturas	São Miguel
BACA0134	*Chroococcales*	*Chroococcaceae*	*Gloeocapsopsis* cf. *dvorakii*	Thermal pool	Benthic	Poça da Silvina	São Miguel
BACA0135	*Nostocales*	*Hapalosiphonaceae*	*Fischerella* sp.	Thermal pool	Benthic	Poças Dona Beja	São Miguel
BACA0136	*Nostocales*	*Chlorogloeopsidaceae*	*Chlorogloeopsis fritschii*	Thermal spring	Benthic	Nascente Água das Quenturas	São Miguel
BACA0137	*Nostocales*	*Microchaetaceae*	*Coleospermum* sp.	Fumarole	Biofilm	Caldeira Grande	São Miguel
BACA0138	*Nostocales*	*Microchaetaceae*	*Coleospermum* sp.	Fumarole	Biofilm	Caldeira do Asmodeu	São Miguel
BACA0139	*Nostocales*	*Microchaetaceae*	*Coleospermum* sp.	Fumarole	Biofilm	Caldeira do Asmodeu	São Miguel
BACA0140	*Nostocales*	*Nostocaceae*	*Cylindrospermum sp.*	Stream	Benthic	Ribeira do Guilherme	São Miguel
BACA0141	*Synechococcales*	*Pseudanabaenaceae*	*Pseudanabaena* sp.	Freshwater Lake	Plankton	Lagoa do Paul	Pico
BACA0142	*Synechococcales*	*Leptolyngbyaceae*	*Leptolyngbya* sp.	Fumarole	Biofilm	Água do Caldeirão	São Miguel
BACA0143	*Nostocales*	*Hapalosiphonaceae*	*Mastigocladus laminosus*	Thermal stream	Benthic	Ribeira Amarela	São Miguel
BACA0144	*Synechococcales*	*Leptolyngbyaceae*	*Leptolyngbya granulifera*	Thermal pool	Benthic	Poça da Silvina	São Miguel
BACA0145	*Synechococcales*	*Leptolyngbyaceae*	*Leptolyngbya gelatinosa*	Thermal stream	Benthic	Ribeira Amarela	São Miguel
BACA0146	*Synechococcales*	*Leptolyngbyaceae*	*Leptolyngbya* sp.	Thermal spring	Benthic	Nascente Água das Quenturas	São Miguel
BACA0147	*Nostocales*	*Tolypothrichaceae*	*Tolypothrix* sp.	Freshwater Lake	Plankton	Lagoa do Fogo	São Miguel
BACA0148	*Chroococcales*	*Microcystaceae*	*Microcystis aeruginosa*	Freshwater Lake	Plankton	Lagoa Azul	São Miguel
BACA0149	*Synechococcales*	*Leptolyngbyaceae*	*Leptolyngbya* sp.	Fumarole	Biofilm	Caldeira do Asmodeu	São Miguel
BACA0150	*Nostocales*	*Hapalosiphonaceae*	*Mastigocladus* sp.	Thermal stream	Benthic	Ribeira Amarela	São Miguel
BACA0151	*Synechococcales*	*Leptolyngbyaceae*	*Leptolyngbya* sp.	Thermal stream	Benthic	Ribeira Amarela	São Miguel
BACA0203	*Synechococcales*	*Leptolyngbyaceae*	*Leptodesmis* sp.	Freshwater Lake	Plankton	Lagoa do Peixe	Pico
BACA0204	*Synechococcales*	*Leptolyngbyaceae*	*Leptolyngbya* sp.	Freshwater Lake	Plankton	Lagoa de São Brás	São Miguel
BACA0223	*Synechococcales*	*Synechococcaceae*	*Anathece minutissima*	Freshwater Lake	Plankton	Lagoa Verde	São Miguel
BACA0224	*Synechococcales*	*Synechococcaceae*	*Cyanobium planeticum*	Freshwater Lake	Plankton	Lagoa Verde	São Miguel
BACA0229	Synechococcales	*Leptolyngbyaceae*	*Leptolyngbya* sp.	Freshwater Lake	Plankton	Lagoa Funda	Flores
BACA0238	Nostocales	*Nostocaceae*	*Nostoc* sp.	Freshwater Lake	Plankton	Lagoa do Congro	São Miguel
BACA0242	Nostocales	*Rivulariaceae*	*Calothrix* sp.	Freshwater Lake	Plankton	Lagoa das Furnas	São Miguel
BACA0293	Nostocales	*Aphanizomenonaceae*	*Aphanizomenon* sp.	Freshwater Lake	Plankton	Lagoa das Empadadas Sul	São Miguel
BACA0294	Nostocales	*Aphanizomenonaceae*	*Aphanizomenon* sp.	Freshwater Lake	Plankton	Lagoa Verde	São Miguel

Table S2. PCR amplifications of MC (mcyC, mcyD, mcyE, mcyG), STX (sxtA, sxtG, sxtH, sxtI) and CYN (cyrB, cyrC) biosynthesis encoding genes and ESI-LC-MS/MS results, concentrations of MC-LR, SXT, and CYN.“+“: amplification of cyanotoxins-producing gene/toxin presence on ESI-LC-MS/MS ; “- “: absence of cyanotoxins-producing amplification/toxin absence on ESI-LC-MS/MS; "*" Detection of ion ADMAdda

		**MC genes**				**STX genes**				**CYN genes**		**ESI-LC-MS**		
**Strain Code**	**Species**	**mcyC**	**mcyD**	**mcyE**	**mcyG**	**sxtA**	**sxtG**	**sxtH**	**sxtI**	**cyrB**	**cyrC**	**MC-LR**	**SXT**	**CYN**
BACA0001	*Nostoc* sp.	-	-	-	-	-	-	-	-	-	-	-	-	-
BACA0002	*Nostoc* sp.	-	-	-	-	-	-	-	-	-	-	-	-	-
BACA0003	*Trichormus* sp.	-	-	-	-	-	-	-	-	-	-	-	-	-
BACA0004	*Rivularia* sp.	-	-	-	-	-	-	-	-	-	-	-	-	-
BACA0005	*Scytonematopsis* sp.	-	-	-	-	-	-	-	-	-	-	-	-	-
BACA0006	*Calothrix* sp.	-	-	-	-	-	-	-	-	-	-	-	-	-
BACA0007	*Kamptonema* sp.	-	-	-	-	-	+	-	-	-	-	-	-	-
BACA0008	*Arthrospira jenneri*	-	-	-	-	-	-	-	-	-	-	-	-	-
BACA0009	*Nostoc paludosum*	-	-	-	-	-	-	-	-	-	-	-	-	-
BACA0010	*Nostoc punctiforme*	-	-	-	-	-	-	-	-	-	-	-	-	-
BACA0011	*Arthrospira jenneri*	-	-	-	-	-	-	-	-	-	-	-	-	-
BACA0012	Tildeniella *sp.*	-	-	-	-	-	-	-	-	-	-	-	-	-
BACA0013	*Nostocaceae*	-	-	-	-	-	-	-	-	-	-	-	-	-
BACA0014	*Pseudanabaena minima*	-	-	-	-	-	-	-	-	-	-	-	-	-
BACA0015	*Pseudanabaena* sp.	-	-	-	-	-	-	-	-	-	-	-	-	-
BACA0016	*Pseudanabaena limnetica*	-	-	-	-	-	-	-	-	-	-	-	-	-
BACA0017	*Microchaete tenera*	-	-	-	-	-	-	-	-	-	-	-	-	-
BACA0018	*Coleospermum* sp.	-	-	-	-	-	-	-	-	-	-	-	-	-
BACA0019	*Cyanobium sp.*	-	-	-	-	-	-	-	-	-	-	-	-	-
BACA0020	*Scytonematopsis* sp.	-	-	-	-	-	-	-	-	-	-	-	-	-
BACA0021	*Coleospermum* sp.	-	-	-	-	-	-	-	-	-	-	-	-	-
BACA0022	*Fortiea* sp.	-	-	-	-	-	-	-	-	-	-	-	-	-
BACA0023	*Nostoc commune*	-	-	-	-	-	-	-	-	-	-	-	-	-
BACA0024	*Planktolyngbya* sp.	-	-	-	-	-	-	-	-	-	-	-	-	-
**BACA0025**	***unidentified species***	-	-	-	-	-	-	-	-	+	+	-	-	+
BACA0026	*Nostoc punctiforme*	-	-	-	-	-	-	-	-	-	-	-	-	-
BACA0027	*Limnothrix* sp.	-	-	-	-	-	-	-	-	-	-	-	-	-
BACA0028	*Tolypothrix* s.l.	-	-	-	-	-	-	-	-	-	-	-	-	-

BACA0029	*Aphanizomenon* sp.	-	-	-	-	-	-	-	-	-	-	-	-	-
BACA0030	*Dolichospermum* sp.	-	-	-	-	-	-	-	-	-	-	-	-	-
**BACA0031**	***unidentified species***	-	-	-	-	-	-	-	-	**+**	**+**	-	-	**+**
BACA0032	*Scytonematopsis sp.*	-	-	-	-	-	-	-	-	-	-	-	-	-
BACA0033	*Coleospermum* sp.	-	-	-	-	-	-	-	-	-	-	-	-	-
BACA0034	*Dolichospermum* sp.	-	-	-	-	-	-	-	-	-	-	-	-	-
BACA0035	*Nostoc* sp.	-	-	-	-	-	-	-	-	-	-	-	-	-
BACA0036	*Nostoc* sp.	-	-	-	-	-	-	-	-	-	-	-	-	-
BACA0037	*Coleospermum* sp.	-	-	-	-	-	-	-	-	-	-	-	-	-
BACA0038	cf. *Isocystis planctonica*	-	-	-	-	-	-	-	-	-	-	-	-	-
BACA0039	*Nostoc* sp.	-	-	-	-	-	-	-	-	-	-	-	-	-
BACA0040	*Coleospermum* sp.	-	-	-	-	-	-	-	-	-	-	-	-	-
**BACA0041**	***Aphanizomenon gracile***	-	-	-	-	**+**	**+**	**+**	**+**	-	-	-	**+**	-
BACA0042	*Nostoc* sp.	-	-	-	-	-	-	-	-	-	-	-	-	-
BACA0043	*Tolypothrix sp.*	-	-	-	-	-	-	-	-	-	-	-	-	-
BACA0044	*Dolichospermum delicatulum*	-	-	-	-	-	-	-	-	-	-	-	-	-
BACA0045	*Limnothrix* sp.	-	-	-	-	-	-	-	-	-	-	-	-	-
BACA0046	*Coleospermum* sp.	-	-	-	-	-	-	-	-	-	-	-	-	-
BACA0047	*Calothrix* sp.	-	-	-	-	-	-	-	-	-	-	-	-	-
BACA0048	*Limnothrix* sp.	-	-	-	-	-	-	-	-	-	-	-	-	-
BACA0049	*Tolypothrix* sp.	-	-	-	-	-	-	-	-	-	-	-	-	-
BACA0050	*Dolichospermum* s.l.	-	-	-	-	-	-	-	-	-	-	-	-	-
BACA0051	*Nostoc* sp.	-	-	-	-	-	-	-	-	-	-	-	-	-
BACA0052	*Nostoc paludosum*	-	-	-	-	-	-	-	-	-	-	-	-	-
BACA0053	*Nostoc* cf. *sphaericum*	-	-	-	-	-	-	-	-	-	-	-	-	-
BACA0054	*Stenomitos* sp.	-	-	-	-	-	-	-	-	-	-	-	-	-
BACA0055	*Microchaete tenera*	-	-	-	-	-	-	-	-	-	-	-	-	-
BACA0056	*Tolypothrix* cf. *helicophila*	-	-	-	-	-	-	-	-	-	-	-	-	-
BACA0057	*Nostoc* sp.	-	-	-	-	-	-	-	-	-	-	-	-	-
BACA0058	*Nostoc* sp.	-	-	-	-	-	-	-	-	-	-	-	-	-
BACA0059	*Nostoc* sp.	-	-	-	-	-	-	-	-	-	-	-	-	-
BACA0060	*Calothrix* sp.	-	-	-	-	-	-	-	-	-	-	-	-	-
BACA0061	*Nostoc* sp.	-	-	-	-	-	-	-	-	-	-	-	-	-

BACA0062	*Microchaete tenera*	-	-	-	-	-	-	-	-	-	-	-	-	-
BACA0063	*Nostoc* sp.	-	-	-	-	-	-	-	-	-	-	-	-	-
BACA0064	*Tolypothrix* sp.	-	-	-	-	-	-	-	-	-	-	-	-	-
BACA0065	*Cyanobacterium* cf. *synechococcoides*	-	-	-	-	-	-	-	-	-	-	-	-	-
BACA0066	*Tolypothrix* sp.	-	-	-	-	-	-	-	-	-	-	-	-	-
BACA0067	*Anabaena* sp.	-	-	-	-	-	-	-	-	-	-	-	-	-
BACA0068	*Nostoc* sp.	-	-	-	-	-	-	-	-	-	-	-	-	-
BACA0069	*Nostoc* sp.	-	-	-	-	-	-	-	-	-	-	-	-	-
BACA0070	*Calothrix* sp.	-	-	-	-	-	-	-	-	-	-	-	-	-
BACA0071	*Planktolyngbya* cf. *limnetica*	-	-	-	-	-	-	-	-	-	-	-	-	-
BACA0072	*Calothrix* sp.	-	-	-	-	-	-	-	-	-	-	-	-	-
BACA0073	*Rivularia* sp.	-	-	-	-	-	-	-	-	-	-	-	-	-
BACA0074	*Tolypothrix* sp.	-	-	-	-	-	-	-	-	-	-	-	-	-
BACA0075	*Aphanothece* sp.	-	-	-	-	-	-	-	-	-	-	-	-	-
BACA0076	*Cyanobium* cf. *plancticum*	-	-	-	-	-	-	-	-	-	-	-	-	-
BACA0077	*Pegethrix* sp.	-	-	-	-	-	-	-	-	-	-	-	-	-
BACA0078	*Phormidium* sp.	-	-	-	-	-	-	-	-	-	-	-	-	-
BACA0079	*Nostoc* s.l.	-	-	-	-	-	-	-	-	-	-	-	-	-
BACA0080	*Calothrix* sp.	-	-	-	-	-	-	-	-	-	-	-	-	-
BACA0081	*Cylindrospermum* sp.	-	-	-	-	-	-	+	-	-	-	-	-	-
BACA0082	*Nostoc* sp.	-	-	-	-	-	-	-	-	-	-	-	-	-
BACA0083	*Rivularia* sp.	-	-	-	-	-	-	-	-	-	-	-	-	-
BACA0084	*Coleospermum* sp.	-	-	-	-	-	-	-	-	-	-	-	-	-
BACA0085	*Coleospermum* sp.	-	-	-	-	-	-	-	-	-	-	-	-	-
BACA0086	*Nostoc* sp.	-	-	-	-	-	-	-	-	-	-	-	-	-
BACA0087	*Nostoc* sp.	-	-	-	-	-	-	-	-	-	-	-	-	-
BACA0090	*Hapalosiphon* sp.	-	-	-	-	-	-	-	-	-	-	-	-	-
BACA0091	*Nostoc* sp.	-	-	+	-	-	-	-	-	-	-	-	-	-
BACA0092	*Nostoc* sp.	-	-	-	-	-	-	-	-	-	-	-	-	-
BACA0093	*Tolypothrix* sp.	-	-	-	-	-	-	-	-	-	-	-	-	-
BACA0094	*Calothrix* sp.	-	-	-	-	-	-	-	-	-	-	-	-	-
BACA0095	*Nostoc* sp.	-	-	-	-	-	-	-	-	-	-	-	-	-
BACA0096	*Nostoc* sp.	-	-	-	-	-	-	-	-	-	-	-	-	-

BACA0097	*Cylindrospermum cf. muscicola*	-	-	-	-	-	-	-	-	-	-	-	-	-
BACA0098	*Tolypothrix* sp.	-	-	-	-	-	-	-	-	-	-	-	-	-
BACA0099	*Nostoc* sp.	-	-	-	-	-	-	-	-	-	-	-	-	-
BACA0100	*Calothrix* sp.	-	-	-	-	-	-	-	-	-	-	-	-	-
BACA0101	*Coleospermum* sp.	-	-	-	-	-	-	-	-	-	-	-	-	-
BACA0102	*Coleospermum* sp.	-	-	-	-	-	-	-	-	-	-	-	-	-
BACA0103	*Nostoc* sp.	-	-	-	-	-	-	-	-	-	-	-	-	-
BACA0104	*Nostoc* sp.	-	-	-	-	-	-	-	-	-	-	-	-	-
BACA0105	*Calothrix* sp.	-	-	-	-	-	-	-	-	-	-	-	-	-
BACA0106	*Tychonema* sp.	-	-	-	-	-	-	-	-	-	-	-	-	-
BACA0107	*Hydrocoryne* cf. *spongiosa*	-	-	-	-	-	-	-	-	-	-	-	-	-
BACA0108	*Lyngbya* cf. *martensiana*	-	-	-	-	-	-	-	-	-	-	-	-	-
BACA0109	*Nostoc* sp.	-	-	-	-	-	-	-	-	+	-	-	-	-
BACA0110	*Coleospermum* sp.	-	-	-	-	-	-	-	-	-	-	-	-	-
BACA0111	*Microchaete bulbosa*	-	-	-	-	-	-	-	-	-	-	-	-	-
BACA0112	*Leptolyngbya gelatinosa*	-	-	-	-	-	-	-	-	-	-	-	-	-
BACA0113	*Phormidium* sp.	-	-	-	-	-	-	-	-	-	-	-	-	-
BACA0114	*Westiellopsis* sp.	-	-	-	-	-	-	-	-	-	-	-	-	-
BACA0116	*Chlorogloeopsis fritschii*	-	-	-	-	-	-	-	-	-	-	-	-	-
BACA0117	*Coleospermum* sp.	-	-	-	-	-	-	-	-	-	-	-	-	-
BACA0118	*Mastigocladus* sp.	-	-	-	-	-	-	-	-	-	-	-	-	-
BACA0119	*Coleospermum* sp.	-	-	-	-	-	-	-	-	-	-	-	-	-
BACA0120	*Coleospermum* sp.	-	-	-	-	-	-	-	-	-	-	-	-	-
BACA0121	*Mastigocladus laminosus*	-	-	-	-	-	-	-	-	-	-	-	-	-
BACA0122	*Mastigocladus laminosus*	-	-	-	-	-	-	-	-	-	-	-	-	-
BACA0123	*Leptolyngbya granulifera*	-	-	-	-	-	-	-	-	-	-	-	-	-
BACA0124	*Coleospermum* sp.	-	-	-	-	-	-	-	-	-	-	-	-	-
BACA0125	*Mastigocladus laminosus*	-	-	-	-	-	-	-	-	-	-	-	-	-
BACA0126	*Chlorogloeopsis fritschii*	-	-	-	-	-	-	-	-	-	-	-	-	-
BACA0127	*Leptolyngbya* cf. *subuliformis*	-	-	-	-	-	-	-	-	-	-	-	-	-
BACA0128	*Mastigocladus laminosus*	-	-	-	-	-	-	-	-	-	-	-	-	-
BACA0129	*Chlorogloeopsis fritschii*	-	-	-	-	-	-	-	-	-	-	-	-	-
BACA0130	*Mastigocladus laminosus*	-	-	-	-	-	-	-	-	-	-	-	-	-

BACA0131	*Chlorogloeopsis fritschii*	-	-	-	-	-	-	-	-	-	-	-	-	-
BACA0132	*Mastigocladus* sp.	-	-	-	-	-	-	-	-	-	-	-	-	-
BACA0133	*Coleospermum* sp.	-	-	-	-	-	-	-	-	-	-	-	-	-
BACA0134	*Gloeocapsopsis* cf. *dvorakii*	-	-	-	-	-	-	-	-	-	-	-	-	-
BACA0135	*Fischerella* sp.	-	-	-	-	-	-	-	-	-	-	-	-	-
BACA0136	*Chlorogloeopsis fritschii*	-	-	-	-	-	-	-	-	-	-	-	-	-
BACA0137	*Coleospermum* sp.	-	-	-	-	-	-	-	-	-	-	-	-	-
BACA0138	*Coleospermum* sp.	-	-	-	-	-	-	-	-	-	-	-	-	-
BACA0139	*Coleospermum* sp.	-	-	-	-	-	-	-	-	-	-	-	-	-
BACA0140	*Cylindrospermum sp.*	-	-	-	-	-	-	-	-	-	-	-	-	-
BACA0141	*Pseudanabaena* sp.	-	-	-	-	-	-	-	-	-	-	-	-	-
BACA0142	*Leptolyngbya* sp.	-	-	-	-	-	-	-	-	+	-	-	-	-
BACA0143	*Mastigocladus laminosus*	-	-	-	-	-	-	-	-	-	-	-	-	-
BACA0144	*Leptolyngbya granulifera*	-	-	-	-	-	-	-	-	-	-	-	-	-
BACA0145	*Leptolyngbya gelatinosa*	-	-	-	-	-	-	-	-	-	-	-	-	-
BACA0146	*Leptolyngbya* sp.	-	-	-	-	-	-	-	-	+	-	-	-	-
BACA0147	*Tolypothrix* sp.	-	-	-	-	-	-	-	-	-	-	-	-	-
**BACA0148**	***Microcystis aeruginosa***	**+**	**+**	**+**	**+**	-	-	-	-	-	-	**+**	-	-
BACA0149	*Leptolyngbya* sp.	-	-	-	-	-	-	-	-	-	-	-	-	-
BACA0150	*Mastigocladus* sp.	-	-	-	-	-	-	-	-	-	-	-	-	-
BACA0151	*Leptolyngbya* sp.	-	-	-	-	-	-	-	-	-	-	-	-	-
BACA0203	*Leptodesmis* sp.	-	-	-	-	-	+	+	-	-	-	-	-	-
BACA0204	*Leptolyngbya* sp.	-	-	-	-	-	+	-	-	-	-	-	-	-
BACA0223	*Anathece minutissima*	-	-	-	-	-	+	-	-	-	-	-	-	-
BACA0224	*Cyanobium plancticum*	-	-	-	-	-	-	-	-	-	-	-	-	-
BACA0229	*Leptolyngbya* sp.	-	-	-	-	-	-	-	-	-	-	-	-	-
BACA0238	*Nostoc* sp.	-	-	-	-	-	-	-	-	-	-	-	-	-
BACA0242	*Calothrix* sp.	-	-	-	-	-	-	-	-	-	-	-	-	-
BACA0293	*Aphanizomenon* sp.	-	-	-	-	-	-	-	-	-	-	-	-	-
BACA0294	*Aphanizomenon* sp.	-	-	-	-	-	-	-	-	-	-	-	-	-

Table S4. ESI-LC-MS/MS analysis parameters for the identification of CYN, MC-LR, and STX.

Cyanotoxin	Retention Time (min)	Precursor ion (m/z)	Product ions	Collision energy	Dwell Time	Polarity	Type
CYN	6.37	416.1	336<br>318<br>274<br>194	20	150	Positive	Target
MC-LR	7.40	995.5	977.28<br>866.33<br>599.16<br>553.18	35	150	Positive	Target
STX	5.75	300.08	282.28<br>265.17<br>241.00<br>240.25<br>221.00<br>204.08<br>186.08	35	150	Positive	Target

Table S3. Sequence identity (%) of 16S rRNA gene fragment between BACA strains and other cyanobacterial sequences available in GenBank (NCBI).

Strain code	Strain	Accession number	Length (bp)	Coveragy (%)	Identity (%)	Closest match (Accession number)
BACA0025	*unidentified species*	MT176703.2	1386	100	96.25	*Cylindrospermum catenatum* CCALA 999 (KF052615.1)
				100	96.18	*Cylindrospermum* sp. NIES-4074 (AP018269.1)
BACA0031	*unidentified species*	MW776414	1137	99	97.18	*Anabaena* sp. 14-VSmolV10 (KT290364.1)
				99	96.83	*Cylindrospermum catenatum* CCALA 999 (KF052615.1)
BACA0041	*Aphanizomenon gracile*	MT176711.3	1425	99	99.58	*Aphanizomenon* cf. *gracile* 271 (AJ293125.1)
				99	98.52	*Aphanizomenon gracile* HEANEY/Camb 1986 140 1/1 (AJ630444.1)
BACA0148	*Microcystis aeruginosa*	MT176750.2	1365	99	99.71	*Microcystis aeruginosa* NIES-90 (LC557463.1)
				100	99.63	*Microcystis aeruginosa* PMC 679.10 (MH892892.1)
