# Supplementary material for: Cyanotoxin Screening in BACA Culture Collection: Identification of New Cylindrospermopsin Producing Cyanobacteria
Source: Toxins (Basel). 2021 Apr 3;13(4):258. doi: 10.3390/toxins13040258 (PMC8065757; doi:10.3390/toxins13040258)
Supplement: Supplementary file 1 [file toxins-13-00258-s001.zip › toxins-1123015- layout sup.docx]

Supplementary Materials： Cyanotoxin Screening in BACA Culture Collection: Identification of New Cylindrospermopsin Producing Cyanobacteria

Rita Cordeiro, Joana Azevedo, Rúben Luz, Vitor Vasconcelos, Vítor Gonçalves and Amélia Fonseca


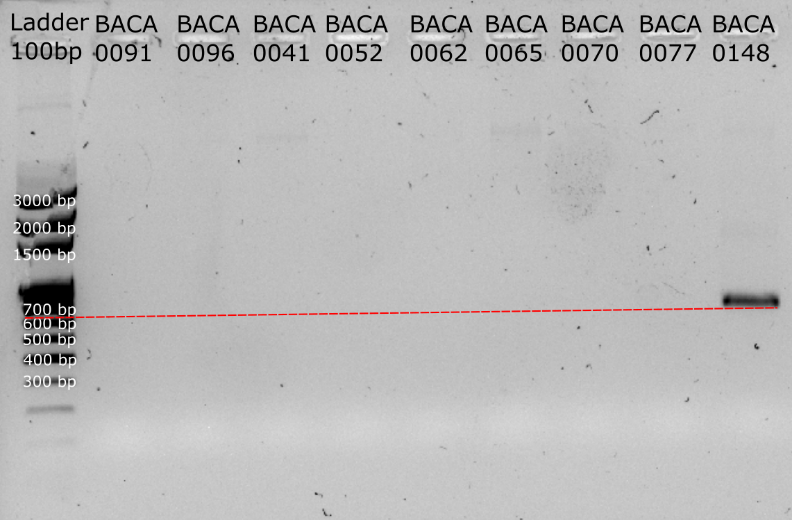

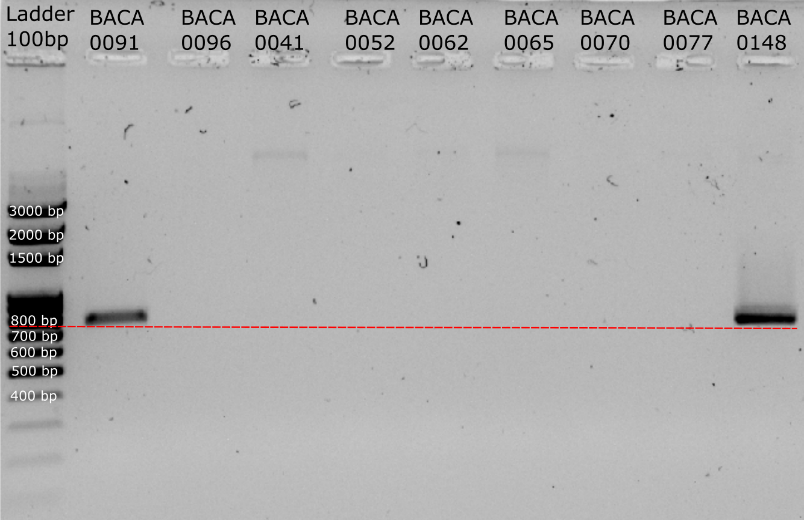


***mcy*C**

***mcy*E**


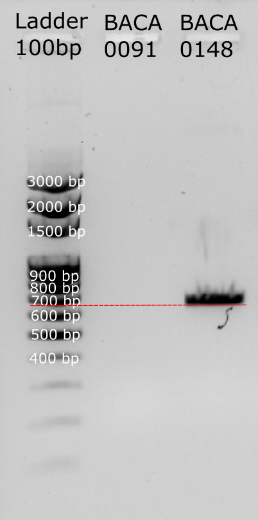

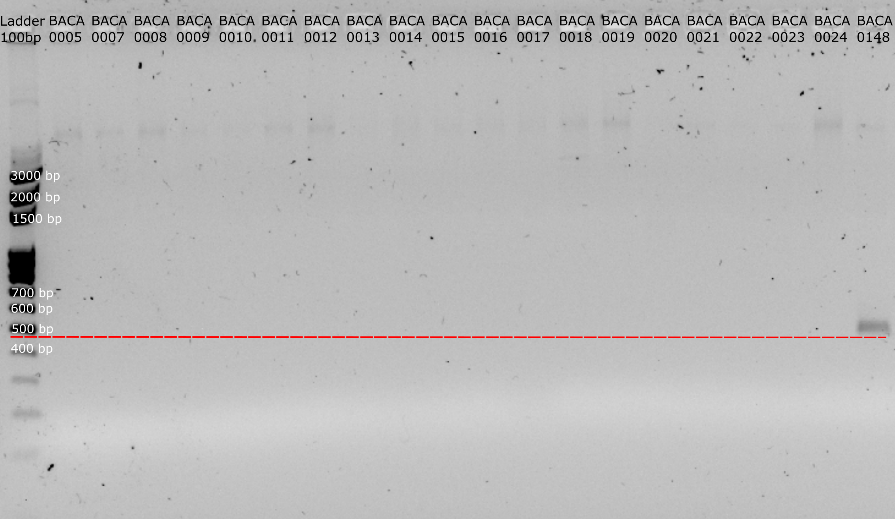


***mcy*D**

***mcy*G**

**Figure 1.** Electrophoresis gel photos of *mcy*C (674 bp), *mcy*D (647 bp), *mcy*E (755 bp) and *mcy*G (425 bp) biosynthesis genes amplifications.


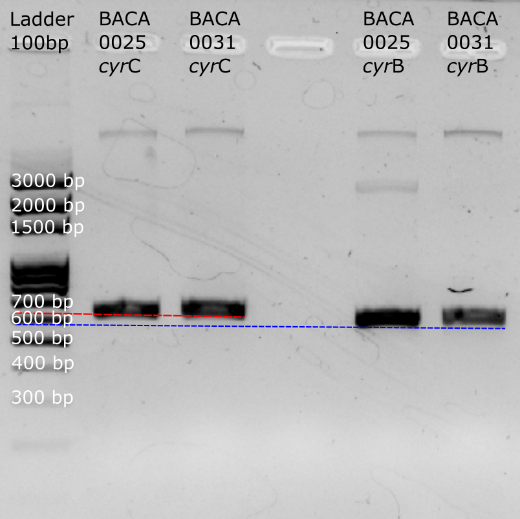

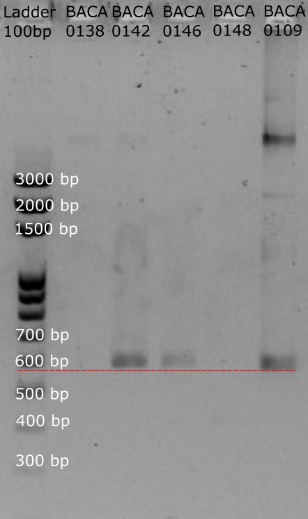


***cyr*B**

***cyr*C*/ cyr*B**

**Figure 2.** Electrophoresis gel photos of *cyr*B (650 bp) and *cyr*C (597 bp) biosynthesis genes amplifications.


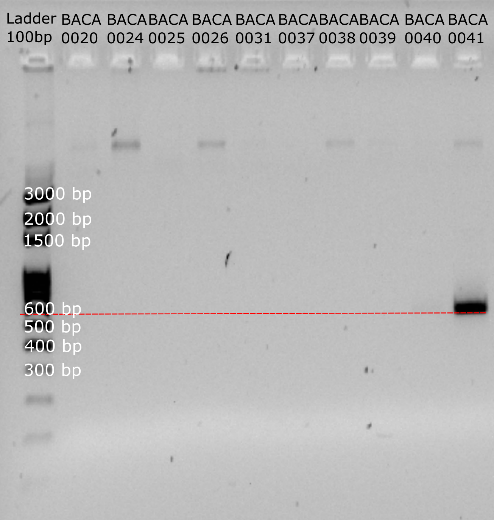

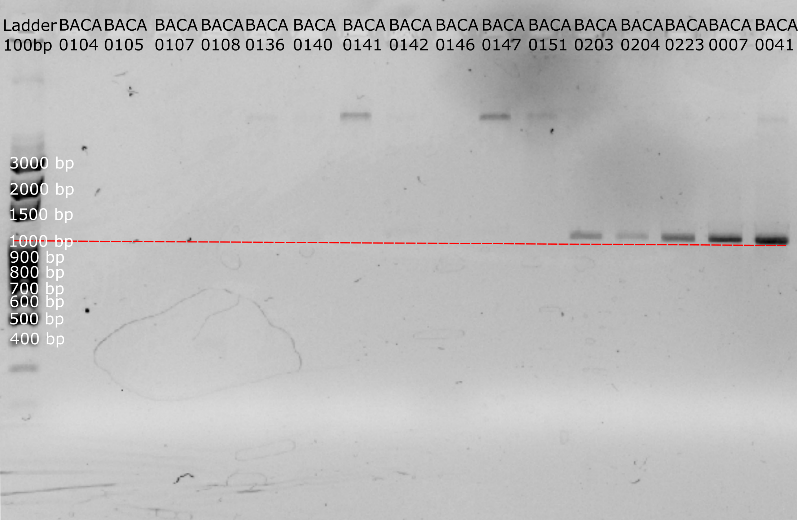


***sxt*A**

***sxt*G**


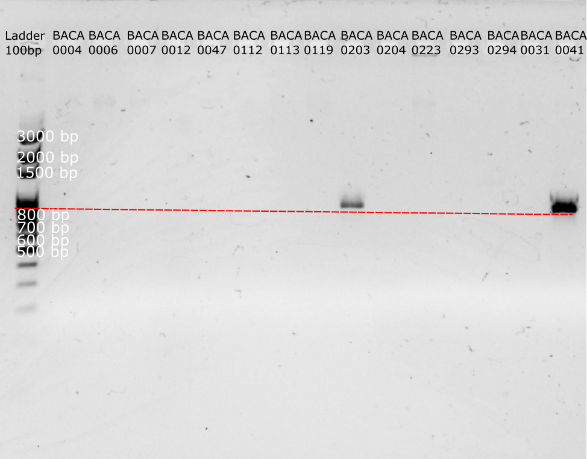

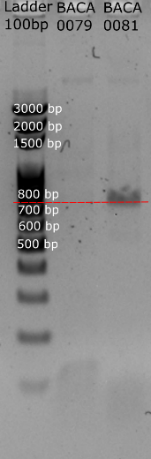

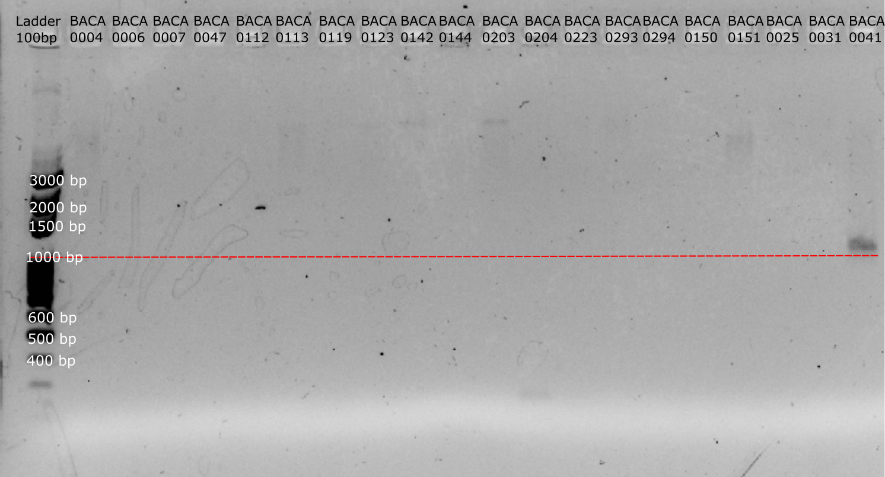


***sxt*H**

***sxt*I**

***sxt*H**

***sxt*I**

**Figure 3.** Electrophoresis gel photos of *sxt*A (602 bp), *sxt*G (893 bp), *sxt*H (812 bp) and *sxt*I (910 bp) biosynthesis genes amplifications.


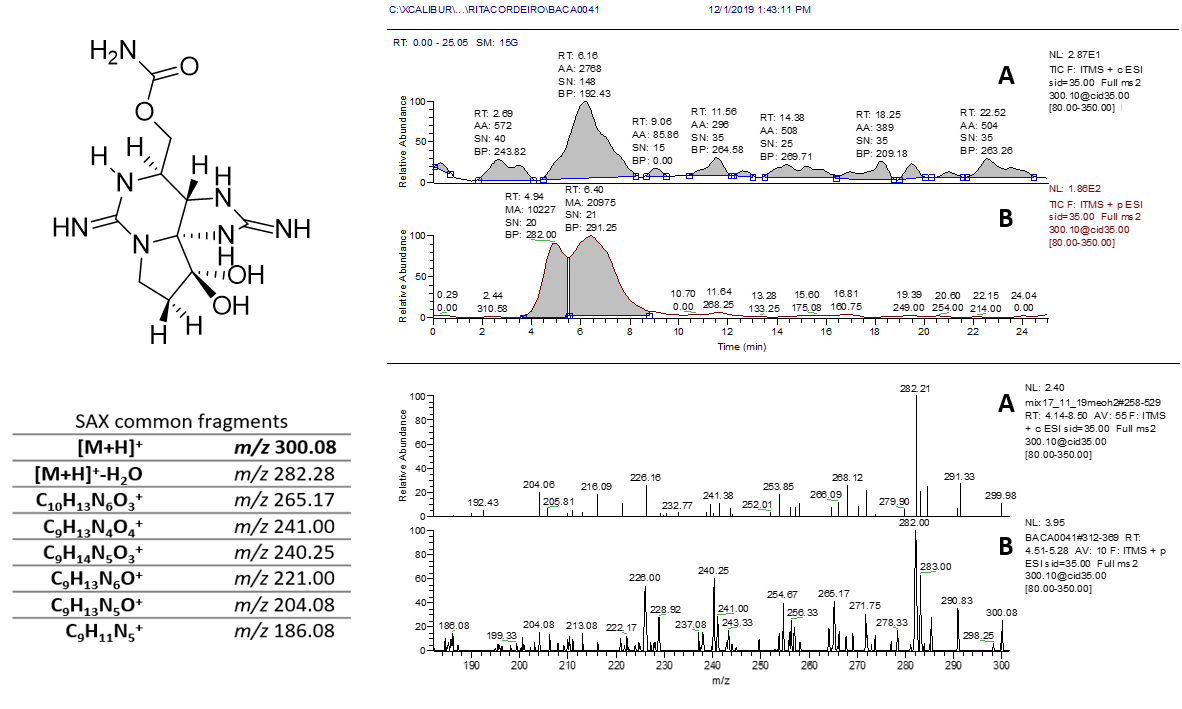


**Figure 4.** Total Ion Chromatograms and spectra of a STX standard solution (A) and sample *Aphanizomenon gracile* BACA0041 (B).


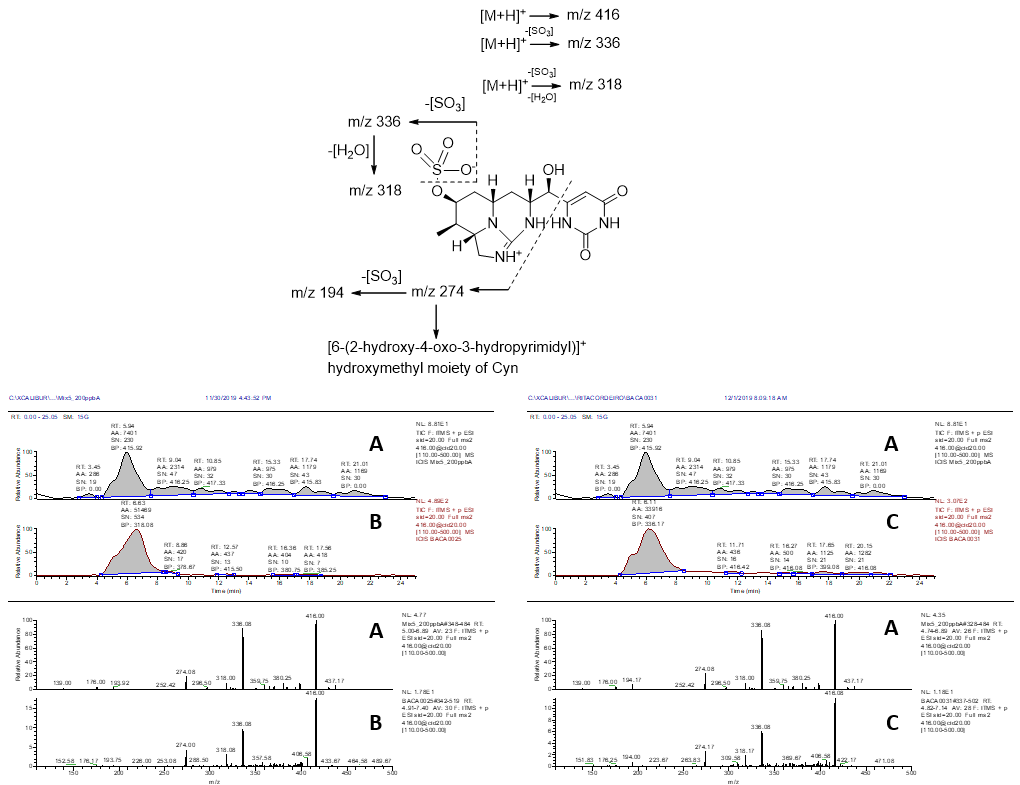


**Figure 5.** Total Ion Chromatograms and spectra of a CYN standard solution (A), sample BACA0025 (B) and sample BACA0031 (C).


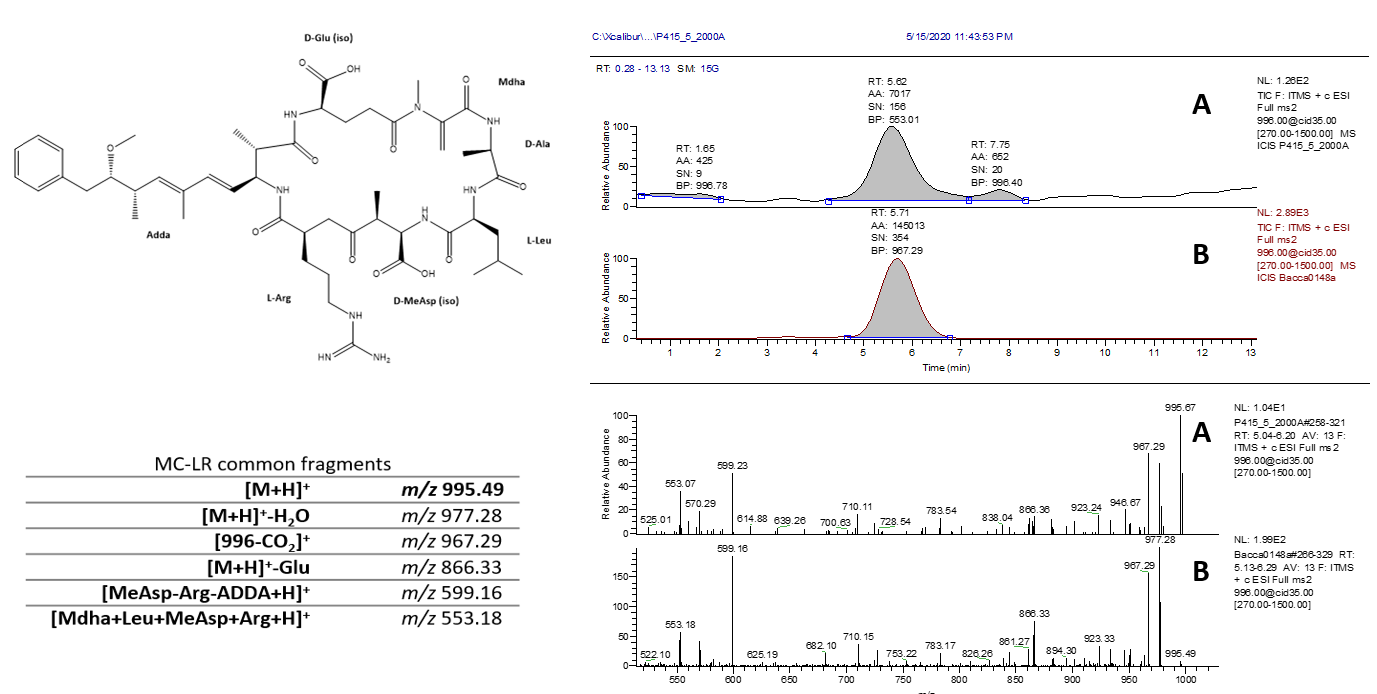


**Figure 6.** Total Ion Chromatograms and spectra of a MC-LR standard solution (A) and sample *Microcystis aeruginosa* BACA0148 (B).
